# Openness to experience and stress responsivity: An examination of cardiovascular and underlying hemodynamic trajectories within an acute stress exposure

**DOI:** 10.1371/journal.pone.0199221

**Published:** 2018-06-18

**Authors:** Páraic S. O’Súilleabháin, Siobhán Howard, Brian M. Hughes

**Affiliations:** 1 School of Psychology, National University of Ireland, Galway, Ireland; 2 Department of Psychology, University of Limerick, Limerick, Ireland; University of Southampton, UNITED KINGDOM

## Abstract

The personality trait openness to experience has been implicated in health, and in particular cardiovascular wellbeing. In a sample of 62 healthy young female adults, the role of openness in cardiovascular responsivity during a stress exposure was examined. Traditionally, methodologies have averaged a stress exposure into a single reading. This may be limited in that it does not consider patterns of cardiovascular adaptation within a stress exposure. Continuous cardiovascular data were reduced to mean 10 second readings, with phases determined through examinations of shifts in responsivity between each 10 second pairing. Analyses revealed a significant linear interaction for openness across the entire exposure for systolic blood pressure, and cardiac output. A significant between-subjects effect for heart rate also emerged. Contrary to their lower counterparts, those highest in openness exhibited an increasingly myocardial hemodynamic response profile throughout the exposure. Comparisons of responsivity suggests adaptive stress response trajectories for those highest in openness. This study also provides evidence that an attenuation of myocardial responsivity may underpin blunted responsivity. This study provides a potential mechanism in reported openness-health associations.

## Introduction

Accumulating research indicates the relevance of the personality trait of openness to experience as a predictor of health outcomes and in particular, cardiovascular wellbeing [1–9]. Openness refers to an individual’s propensity to be open to a variety of experiences, with a need to enlarge and examine experience [10, 11]. A meta-analysis, in addition to a 10-year follow-up study has found that higher openness is protective with respect to all-cause mortality [2, 8]. Openness has also been found to be implicated in cardiovascular wellbeing, such that higher openness has been observed as a protective factor. More specifically, data pertaining to a 10.5 year follow-up study found that openness was associated with coronary heart disease (CHD), with higher openness being observed as being an independent protective risk factor [12]. Openness was found to be the sole personality trait predictive of CHD [12]. A further study drawn from the Health and Retirement Study found that higher openness reduced the odds of diagnosis of multiple cardiovascular health associations; namely stroke by 31%, high blood pressure by 29%, and heart conditions (myocardial infarction, CHD, angina, cardiac heart failure, or other heart problems) by 17% [13]. Collectively, the aforementioned authors concluded potential mechanisms which may account for these associations as being unclear. Openness constitutes a trait which accounts for an individual’s motivation and receptiveness for experiences. As such, a naturally selected trait such as openness should be of crucial importance to an individual’s responsivity to stress experiences. As suggested by Ó Súilleabháin and colleagues [14] persons highest in openness should possess the required ability to stimulate short-term stress responsivity, while demonstrating an ability to habituate across time.

Metabolically inappropriate cardiovascular reactivity (difference between stress elevation and baseline) to psychological stress is thought to disrupt homeostasis in ways which are detrimental to health [15]. Indeed, as reviewed by Phillips and Hughes [16], extensive prospective and cross-sectional research supports the association between heightened cardiovascular reactivity and increased risk of cardiovascular disease (CVD); including hypertension, atherosclerosis, myocardial infarction (MI), increased left ventricular mass (LVM), and CHD mortality [17–26]. While most research has examined the adverse implications of elevated cardiovascular responsivity, significant caveats have emerged [27]. While elevated cardiovascular responsivity to stress over prolonged periods can be considered as leading to negative outcomes, they may also be adaptive in the short-term when responding to acute stress [27]. As highlighted by Hughes [27], research has observed that acute stress can stimulate immune effectiveness, and that cardiovascular stress responding is positively associated with enhanced immune responding [28]. The potential negative implications of sustained responsivity may also be diminished due to the habituation of responsivity across time (e.g. [29]). Indeed, when individuals are presented with similar stress exposures, patterns of cardiovascular stress adaptation have been observed (e.g., [29,30]). Recent research has also observed that patterns of cardiovascular adaptation can occur across a change in stress exposures [14].

Research examining the potential associations between openness and cardiovascular stress responding is limited. Recently Ó Súilleabháin and colleagues [14] reported that higher openness stimulates short-term stress responsivity, while ensuring cardiovascular habituation to change in stress across time. Further research has also reported higher openness as associated with lesser heart rate (HR) reactivity across repeated social stress exposures, in addition to lesser SBP reactivity to the repeated stress exposure [31]. In addition, Williams and colleagues [32] found persons higher in openness to exhibit lower SBP and DBP to stress tasks involving the recall of stressful experiences. Further research has reported a positive association between openness and HR reactivity in a midlife sample [33]. These aforementioned studies, and indeed wider stress research have traditionally employed methodologies which quantify a stress response as an averaged reading across an entire stress exposure. While this strategy has provided significant contributions, it may be limited in that it does not consider trajectories within an exposure.

The examination of responsivity during a stress exposure has the potential to uncover highly relevant associations which may otherwise be masked by reducing the entire stress experience to a single measurement. In other words, responsivity which may appear elevated or diminished when reducing the exposure to a single measurement may mask both adaptive and maladaptive trajectories which have significant health implications. As such, the present study sought to examine if openness is associated with shifts in cardiovascular responsivity within a stress exposure. Given the potential relevance of openness as a facilitator for adjustment to stress experiences, the present study examined if openness is related to cardiovascular and underlying hemodynamic response patterns during a stress exposure.

## Methods

### Participants

Participants were healthy female college students (*N* = 62; *M* ± *SD* = 19.15 ± 1.32 years; range = 17–24 years; BMI, *M* ± *SD* = 22.96 *±* 2.95 kg/m^2^). Females were examined due to a lack of availability of a biometrically comparable sample of males within the college population in psychology. Each participant provided written informed consent. Persons below the age of 18 years provided parental consent to participate. Given the accumulative nature of the health associations implicated with openness, the examination of healthy individuals was of importance. As such, participants reported not consuming cardioactive medication or suffering from any cardiovascular illness. Sample size is comparable to those of similar research (e.g., [34]).

### Psychometric assessment

Openness (*M* ± *SD* = 27.60 *±* 7.60) was assessed using the NEO Five Factor Inventory (NEO FFI-3; [11]). Current mean openness levels are within the average range for female adults within published norms [35]. Openness reliability alpha (Cronbach’s) in the sample was excellent (α = .82).

### Physiological assessment

A Finometer (Finapres Medical Systems BV, BT Arnhem, The Netherlands) was used to examine cardiovascular function. The Finometer is a continuous hemodynamic monitor that assesses beat-to-beat blood pressure and heart rate. Using the volume-clamp method [36], the Finometer obtains measurements by photophethysmography via a finger cuff. The Finometer has been shown to successfully assess accurate blood pressure measurements in a variety of samples [37, 38]. Beat-to-beat measures were obtained continuously at a sampling rate of 200 Hz. While the Finometer maintains a low sensitivity to motion artifacts, the sensor is also securely fastened to each participants wrist to further minimise potential artifacts. Hand position was maintained at the correct level throughout. Calibration using the Finometer’s patented Return-to-Flow technology was conducted on each participant. This results in the Finometer achieving a standard of absolute blood pressure measurement for each participant in meeting the validation criteria of both the British Hypertension Society, and the Association for the Advancement of Medical Instrumentation [37].

### Procedure

In order to limit the impact of circadian rhythms testing took place between 09:00 and 13:00. In addition, in order to limit the impact of any environmental variation on physiological responsivity [39], all testing took place in the same laboratory. Participants received instructions not to partake in exercise for 2 hours prior to attending and to not consume any caffeinated products. Participants were greeted by the researcher on arrival, and their height and weight were digitally recorded. Following being seated in a comfortable chair, the Finometer was attached to the middle finger of their non-dominant hand. A 30-minute acclimatization period followed prior to the commencement of the experimental protocol. To facilitate relaxation and establishment of cardiovascular baseline levels, participants were provided with non-emotive reading material [40]. Following this period, the formal protocol commenced with a baseline period of 10 minutes; followed by a 5-minute exposure to a mental arithmetic task. The mental arithmetic stressor employed was presented on a computer screen where participants were required to solve on-screen subtraction problems to which participants entered their responses via a keyboard. This is a standard stress procedure which has been repeatedly employed in existing literature (e.g. [14]). Given the associations between openness and an ability to perform tasks (e.g., [41]), the task controlled for mathematical ability where subtraction items became more challenging or easier when three consecutive correct/incorrect responses were entered. This has also been shown to be effective in the maintenance of engagement and stressfulness during cardiovascular stress research [42–44]. Beat-to-beat measures of systolic blood pressure (SBP), diastolic blood pressure (DBP), heart rate (HR), cardiac output (CO), and total peripheral resistance (TPR) were obtained continuously throughout.

### Phase reduction

SBP, DBP, HR, CO and TPR continuous measurements were computed as the mean 10 second readings throughout the stress exposure. In order to determine potential phases of shifts in responsivity; paired samples *t*-tests were conducted between each incremental 10 second period for each cardiovascular parameter. Thus, SBP, DBP, HR, CO and TPR were each examined individually for changes between each 10 second pairing. It was determined that a phase would be constituted if the paired samples *t*-test was significant.

**Fig 1:**
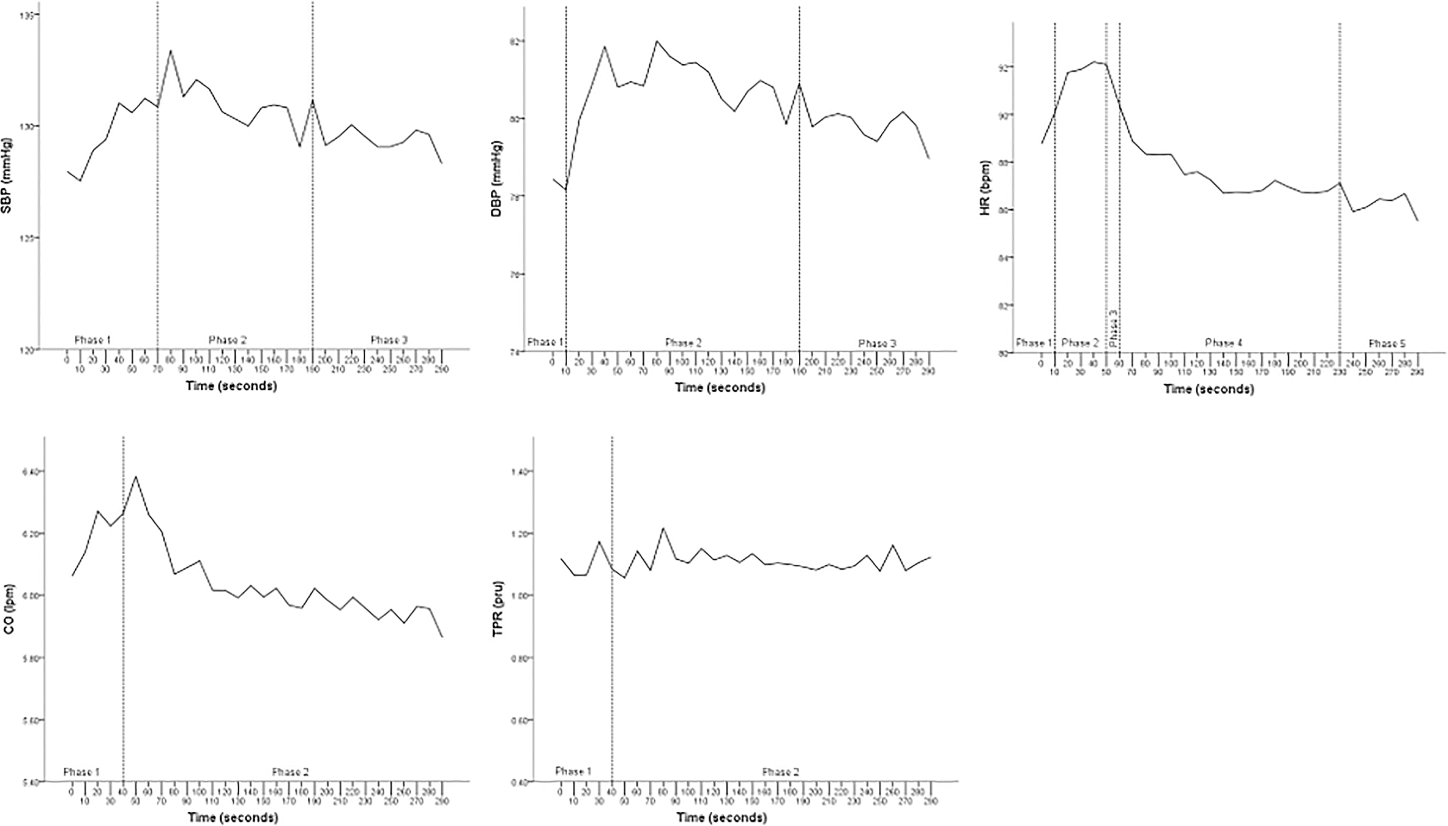
SBP, DBP, HR, CO and TPR mean function across the entire stress exposure delimited by created phases.

SBP, paired samples *t*-tests *t*(61) = -2.154, *p* = .035 between 70 and 80 seconds; *t*(61) = 2.024, *p* = .047 and 190 and 200 seconds. DBP, *t*(61) = -2.167, *p* = .034 between 10 and 20 seconds; *t*(61) = 2.425, *p* = .018 between 190 and 200 seconds. HR, *t*(61) = -3.084, *p* = .003 between 10 and 20 seconds; *t*(61) = 2.855, *p* = .006 between 50 and 60 seconds; *t*(61) = 3.317, *p* = .002 between 60 and 70 seconds; *t*(61) = 2.703, *p* = .009 between 230 and 240 seconds. CO, *t*(61) = -2.528, *p* = .014 between 40 and 50 seconds. TPR, *t*(61) = 2.95, *p* = .005 between 40 and 50 seconds. For graphical illustration of created phases, see Fig 1. Excellent internal reliability consistency for each measure was observed (Cronbach’s α; SBP = .92, DBP = .90, HR = .96, CO = .96, and TPR = .89).

As the multiple comparisons may increase the familywise error rate, the established phases were examined using ANOVA for each parameter. Within-subjects ANOVA confirmed a main effect for phase, for SBP, Wilk’s λ = .88, *F*(2, 60) = 4.05, *p* = .022, DBP, Wilk’s λ = .73, *F*(2,60) = 11.00, *p* < .001, and HR, Wilk’s λ = .66, *F*(4, 58) = 7.45, *p* < .001. Paired-sample *t*-test confirmed a difference in the two CO phases, *t(61) = 2*.*18*, p = .034, but not the two TPR phases, *p* = .792, indicating clear difference across the established phases for all parameters except TPR.

### Overview of analyses

To examine the potential impact of openness throughout the stress exposure, a series of mixed factorial ANCOVAs were conducted using PASW Statistics 22.0 (SPSS Inc., Chicago, IL; S1 File). The within-subjects variable was phase, namely the computed mean of the identified phases for each cardiovascular variable. For each corresponding cardiovascular parameter, the first data points at the commencement of the exposure and relevant baseline period were included as covariates. Given the inclusion of both aforementioned covariates, openness was examined through its inclusion as a between-subjects factor in tertiles; lowest (*n* = 22), middle (*n* = 20), and highest (*n* = 20). In other words, given the importance of including both covariates due to their potential in driving response trajectories, openness was examined as a between-subjects factor for clarity of interpretation. This approach to examining personality traits has been frequently used and indeed is recommended to guard against the violation of the homogeneity of regression that would likely occur should openness be treated as a covariate [45, 46]. Both covariates were significantly correlated with the dependent variables in each instance, thus reducing the potential of hampering power by their inclusion through reducing degrees of freedom. Significant findings are graphically represented by tertiles of openness and reactivity trajectories following stress commencement. Where sphericity assumptions were violated, Greenhouse-Geisser results were reported. The assumption of homogeneity of variance was not violated. Partial η^2^ values of .04, .25, and .64 were taken to represent small, medium and large effect sizes respectively [47, 48]. Descriptive statistics for all parameters are outlined in Table 1.

**Table 1:** Mean and standard deviations for all cardiovascular measures across each phase of the experiment.

	Stress Exposure									
	Phase									
	Phase 1		Phase 2		Phase 3		Phase 4		Phase 5	
	Mean	*SD*	Mean	*SD*	Mean	*SD*	Mean	*SD*	Mean	*SD*
**SBP (mmHg)**	129.69	10.55	131.01	13.06	129.34	12.90	-	-	-	-
**DBP (mmHg)**	78.30	8.80	80.94	8.37	79.78	8.32	-	-	-	-
**HR (bpm)**	89.42	11.45	91.99	14.58	90.40	14.40	87.33	11.35	86.18	10.30
**CO (lpm)**	6.19	1.58	6.02	1.36	-	-	-	-	-	-
**TPR (pru)**	1.10	.48	1.11	.40	-	-	-	-	-	-

## Results

### Elicitation of stress response

A series of one-way repeated-measures ANOVAs were conducted to examine if the task employed was successful in eliciting a stress response. Consistent with the increase from baseline to task, significant linear effects were observed across time (SBP, *F*(1, 61) = 111.14, *p* < .001, partial η^2^ = .646; DBP, *F*(1, 61) = 149.42, *p* < .001, partial η^2^ = .71; HR, *F*(1, 61) = 49.68, *p* < .001, partial η^2^ = .449; CO, *F*(1, 61) = 50.596, *p* < .001, partial η^2^ = .453; TPR, *p* = .221).

### Cardiovascular responses and personality

#### SBP, DBP, HR

**Fig 2:**
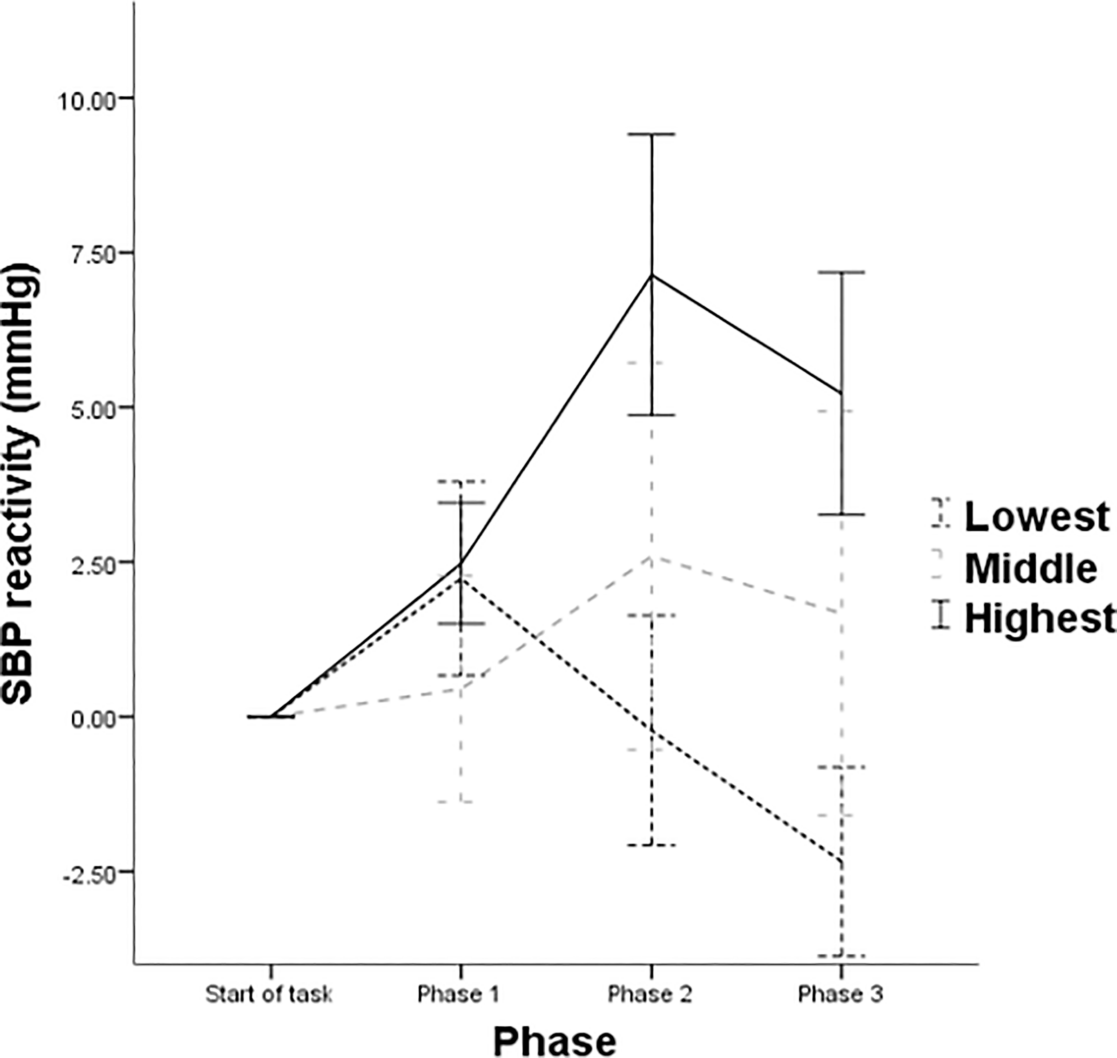
Patterns of mean SBP function across each phase of the experiment by tertiles of openness. Note: Error bars denote ± 1 standard error of the mean.

For SBP, while there was no significant within-subjects effects for phase (*p* = .442), a significant phase × openness interaction effect was observed *F*(3.12, 88.85) = 4.40, *p* = .006, partial η^2^ = .134. Examination of within-subjects contrasts revealed a significant phase × openness linear interaction *F*(2, 57) = 5.06, *p* = .010, partial η^2^ = .151 (see Fig 2), with those highest in openness displaying an elevation of responsivity compared to their lower counterparts. The observed between-subjects effect was not significant (*p* = .084).

For DBP, no significant within-subjects effect for phase or phase × openness interaction emerged (all *p*s > .077). No significant within-subjects contrasts effects emerged (all *p*s > .064). Additionally, a significant between-subjects effect was also not observed (*p* = .093).

**Fig 3:**
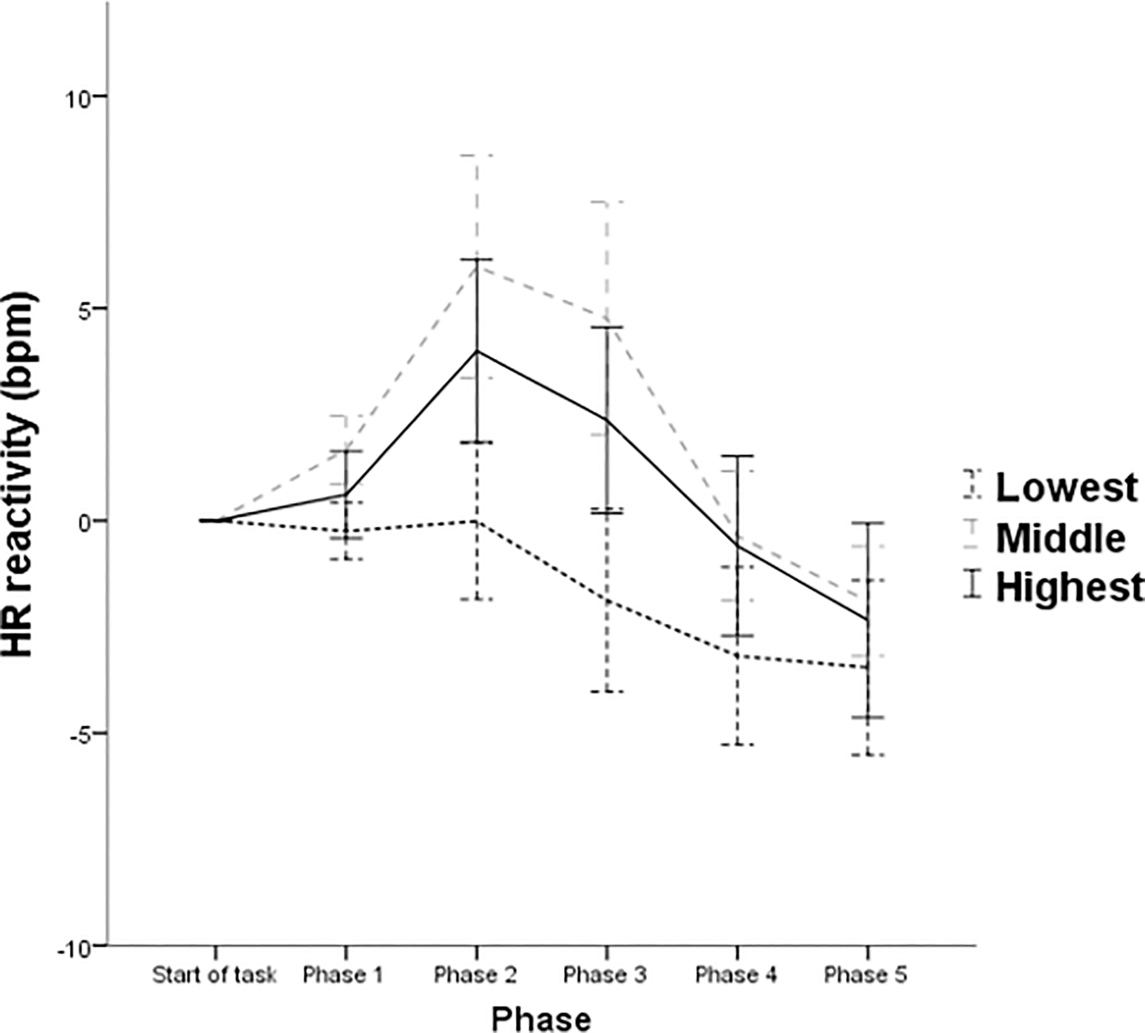
Patterns of mean HR function across each phase of the experiment by tertiles of openness. Note: Error bars denote ± 1 standard error of the mean.

For HR, the within-subjects main effect for phase and phase × openness interaction did not emerge as significant (*p* = .165). In addition, no significant within-subjects contrasts phase × openness effect was observed (all *p*s > .089). However, a significant between-subjects effect emerged for openness *F*(2, 57) = 3.33, *p* = .043, partial η^2^ = .105 (see Fig 3). Pairwise comparisons revealed that the effect for openness reflected higher HR for those in the middle tertile of openness compared to their lower counterparts (mean difference ± SE = 5.11 ± 1.99 bpm; *p* = .039 [Bonferroni corrected]). No significant pairwise comparison was observed between persons higher in openness and their lower (*p* = .47) and middle (*p* = .83) counterparts.

#### CO, TPR

**Fig 4:**
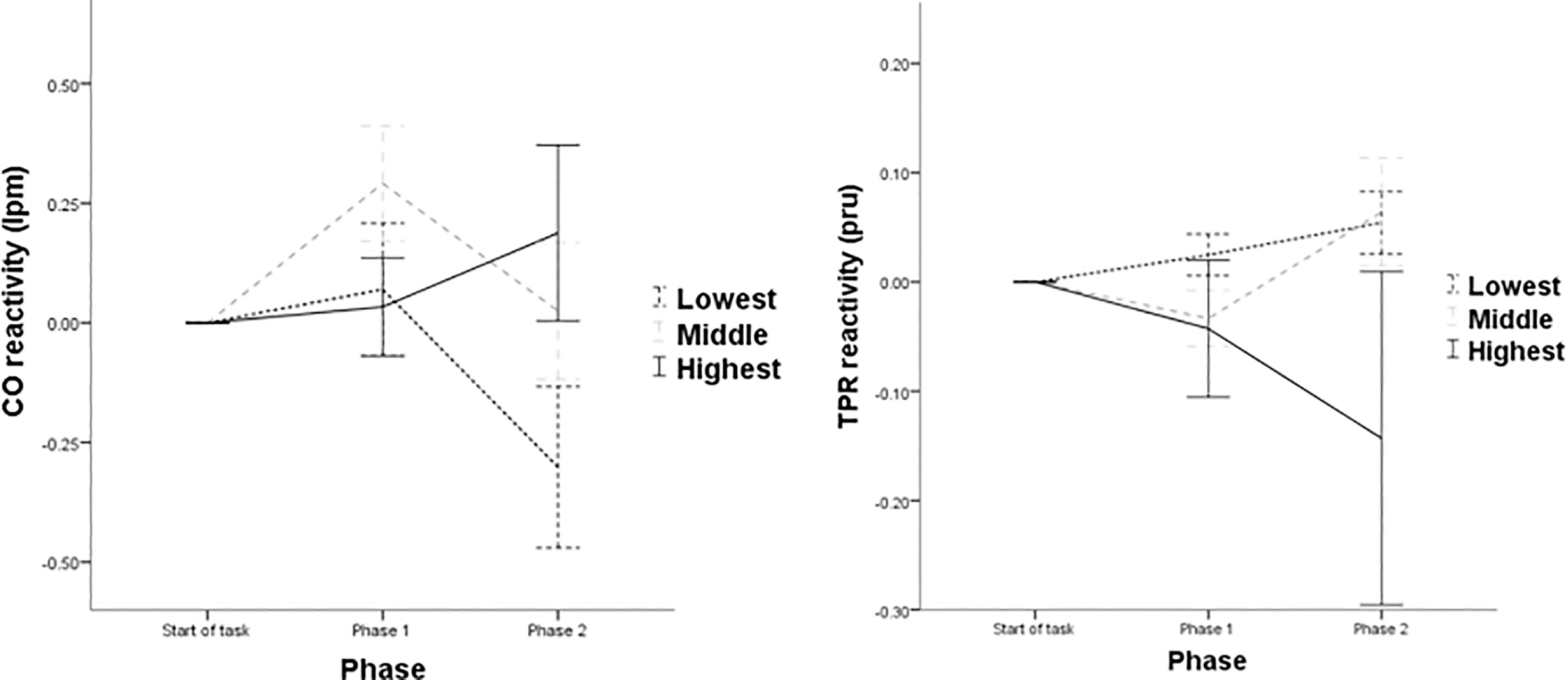
CO and TPR reactivity across both phases of the experiment by tertiles of openness. Note: Error bars denote ± 1 standard error of the mean.

For CO, the main within-subjects effect for phase did not emerge as significant (*p* = .179). A significant phase × openness within-subjects effect was observed *F*(1, 57) = 4.15, *p* = .021, partial η^2^ = .127. Analyses of the within-subjects contrasts effects revealed a significant phase × openness linear interaction *F*(2, 57) = 4.15, *p* = .021, partial η^2^ = .127, with those in the middle and lowest tertile of openness exhibiting an attenuation of responsivity across phases (see Fig 4). No significant between-subjects effect emerged (*p* = .313).

For TPR, a significant main within-subjects effect, phase × openness within-subjects effect, phase × openness within-subjects contrasts effect, or between-subjects effect did not emerge (all *p*s > .115). As previously outlined, TPR phases were not confirmed with the within-subjects ANOVA.

## Discussion

The present study provides evidence that openness is relevant to consider when seeking to predict responsivity during an acute stress exposure. Openness was observed as being associated with SBP throughout the exposure. Persons highest in openness were found to exhibit what appears to represent adaptive response trajectories to the stress experience. Indeed, this is line with existing research suggesting that higher openness facilitates short term stress responsivity while ensuring habituation across time [14]. As previously highlighted, existing literature suggests healthful associations with short-term stress responsivity [27, 28]. Comparatively, those within the middle and lowest tertiles were found to exhibit low SBP stress responsivity. Compared to the remaining tertiles, individuals lowest in openness were also found to display low HR responsivity. Openness was also found to be relevant within CO response trajectories throughout the stress exposure. Persons highest in openness exhibited an increasing CO hemodynamic response profile which is thought to constitute an adaptive response profile. Comparatively, those within the middle and lowest tertiles both exhibited an attenuating trend from CO responsivity during the stressor. Collectively, the aforementioned findings suggest that the immediate adjustment to stress was tolerated in a more adaptive manner by those in the highest tertile of openness.

As discussed previously, the motivation and capacity to be receptive to experiences is central to openness. As such, it is unsurprising that those highest in openness produce a stimulation of responsivity to a stressor requiring active engagement. In other words, persons highest in openness appear to possess a flexibility to responds to the presented stressful experience. Interestingly, a distinct difference between those highest in openness and their lower counterparts with respect to their hemodynamic profile (the reciprocal relationship between CO and TPR) emerged. Those highest in openness were observed to display an increasingly myocardial (CO) dominated hemodynamic profile during the stress exposure. Both of the remaining tertiles of openness were observed to exhibit a differing profile, characterised by an attenuation of myocardial responsivity. A myocardial dominated profile is thought to be more adaptive and less atherosclerotic than a vascular-dominated profile [49]. As such, the observed tendency for those highest in openness to mount an increasingly myocardial response profile within the stress exposure may indicate a protective effect to the more negative health associated vascular orientation (TPR). Indeed, the protective implications of higher openness in cardiovascular health associations have been documented [13].

As previously discussed, research investigating cardiovascular responses to stress typically examine stress as an averaged reading across an entire exposure. While research have examined cardiovascular trajectories during acute stress (e.g. [50, 51]), this research is the first to examine the association between a stress exposure and personality in this manner. It was unclear what, if any, associations may emerge once the exposure was magnified. The present findings significantly add to existing literature examining openness and stress responsivity. It highlights that higher openness appears to facilitate a distinctly adaptive cardiovascular response profile during a stress exposure. The adaptive and healthful value of adequately responding to acute stress in the short-term has been previously outlined [28]. Aside from implications observed with openness, these findings make a significant contribution to broader research examining cardiovascular stress responses. The present study has observed that a personality trait is associated with differing cardiovascular and hemodynamic response trajectories can be observed within a stress exposure. Indeed, the more adaptive myocardial-dominated response can be seen to increase for some (those highest in openness), while attenuating for others (those in the middle and lowest tertiles of openness). As suggested by James and colleagues [52], differing hemodynamic response profiles may result in blunted blood pressure responsivity, such that blunting may be reflective of vascular response tendencies. Indeed, the present study found that those in the lowest tertile of openness who could be characterised as displaying blunted SBP responsivity exhibited a decreasingly myocardial response. Thus, the blood pressure responses and underlying increasingly-myocardial responsivity during a stress exposure would appear to signify an adaptive response for those highest in openness. Contrarily, the low SBP responses, in addition to underlying attenuation of myocardial responsivity for their lower counterparts, may indicate a maladaptive response profile which may be implicated in negative health associations [53].

While the present study consists of a number of strengths, limitations must be noted. While the present sample size is comparable to existing research, employing a larger sample size may have detected smaller effects. Future research also needs to employ a biometrically comparable sample of males given research has also implicated differing stress responsivity across sexes. In addition, the incorporation of further measures of stress and task engagement both prior to and following the task would add further value to future research. The examination of various other stress tasks would also benefit the current literature, as would rates of recovery following stress exposure. Future research may also benefit from the investigating various approaches to continuous stress data, such as through the employment of correlational or regression statistical procedures. While the lack of significance with respect to TPR is consistent with existing research indicating active stressors eliciting myocardial dominated responsivity, it would be worthwhile for future research to examine differing stress exposure types. Particularly those of theoretical relevance to the personality trait or individual difference under examination [54]. Future research would benefit from examining the effects of differing personality traits and coping mechanisms on cardiovascular trajectories within an acute stress exposure [55]. In addition, recent research suggests cardiovascular mechanisms may be responsible for the association between personality and mortality [56], and as such future research should also seek to examine the effects of personality across stress responsivity and resulting mortality effects. Future research may also find wish to examine how phases of shifts in responsivity in hemodynamic may directly map to shifts in changes in both blood pressure and heart. This would provide further linkages between underlying hemodynamic mechanisms, and both blood pressure and heart rate.

The results from the present study demonstrate that openness is associated with cardiovascular and underlying hemodynamic response trajectories within a stress exposure. In line with theoretical implications of this traits relevance in experiences, persons highest in openness appear to possess an ability to respond in an adaptive manner to a newly presented stress experience. Aside from highlighting the relevance of examining response trajectories within a stress exposure, the observed findings do suggest a distinct flexibility for those highest in openness. Personality traits have been suggested as having evolved as strategies to solve adaptive problems, such that scoring highly on a trait is adaptive in one context and not in another [57]. In the case of openness, being higher in openness may facilitate the organism in responding to a newly presented stressor in ways which are adaptive. Certainly, the present study would suggest persons lower in openness as not being characterised by a required stimulation of physiological responsivity within the stress experience. In addition to highlighting the importance for future stress research to examine shifts in responsivity during acute stress, the present study implicates openness in the context of responsivity during a stress exposure.

## Supporting information

S1 File Dataset.
(SAV)
